# A Complex Case of Obstructive Jaundice in a Septuagenarian: Diagnostic Challenges and Therapeutic Strategies

**DOI:** 10.7759/cureus.64598

**Published:** 2024-07-15

**Authors:** Sana Anwar, Ali Afaq Rasool Malik, Ali Hamza, Muhammad Salman Shahid, Muhammad Subhan, Ruqiya Bibi

**Affiliations:** 1 Internal Medicine, Lugansk State Medical University, San Antonio, USA; 2 Medicine, King Edward Medical University, Lahore, PAK; 3 Internal Medicine, Allama Iqbal Medical College, Lahore, PAK; 4 Medicine and Surgery, Jinnah Hospital Lahore, Lahore, PAK; 5 Demonstrator, Amna Inayat Medical College, Allama Iqbal Medical College, Lahore, PAK

**Keywords:** emergency pancreaticoduodenectomy, liver function test (lft), whipple procedure, pet ct scan, pediatric gastroenterology, diagnostic and therapeutic ercp, adenocarcinoma, common bile duct (cbd), ampullary carcinoma, obstuructive jaundice

## Abstract

Obstructive jaundice occurs when an obstruction in the bile duct system prevents bile from flowing from the liver into the intestine, accumulating bilirubin in the blood. This condition can result from various causes, including gallstones, tumors, or inflammation of the bile ducts. The management of obstructive jaundice depends on the underlying cause (malignant obstructions such as cholangiocarcinoma or pancreatic cancer), indicating the need for surgical intervention. The Whipple procedure (pancreaticoduodenectomy) is the standard curative approach for resectable distal common bile duct (CBD) adenocarcinoma. Doctors usually recommend adjuvant chemotherapy to reduce the risk of recurrence. We report the case of a 70-year-old male with a history of untreated hypertension, type 2 diabetes, and long-term smoking, who presented with classic signs of obstructive jaundice, including yellowing of the eyes, itching, right upper quadrant pain, and intermittent fevers. Laboratory findings revealed elevated inflammatory markers, bilirubin, liver enzymes, and leukocyte count, indicative of an inflammatory and obstructive biliary condition. Imaging studies confirmed a distal CBD stricture, including abdominal ultrasound, computed tomography scans, and endoscopic retrograde cholangiopancreatography (ERCP). Brush cytology obtained during ERCP revealed a well-differentiated adenocarcinoma of the distal CBD. The patient's treatment plan included preoperative optimization, surgical resection via the Whipple procedure, and postoperative adjuvant therapy. This case emphasizes the importance of a thorough diagnostic workup and a multidisciplinary treatment strategy in managing complex cases of obstructive jaundice in the elderly, highlighting the need for personalized care to achieve optimal outcomes.

## Introduction

Obstructive jaundice in the elderly presents a significant diagnostic and therapeutic challenge, often complicated by the presence of multiple comorbid conditions that can obscure or mimic the symptoms of biliary obstruction [1]. The incidence of obstructive jaundice increases with age due to a higher prevalence of underlying conditions such as gallstones, malignancies, and biliary tract diseases [2]. In elderly patients, the clinical presentation can be atypical, and the presence of comorbidities such as hypertension (HTN), diabetes mellitus (DM), cardiovascular disease (CVD), and a history of smoking further complicates both diagnosis and management [3]. Obstructive jaundice occurs when an obstruction in the bile duct system prevents bile from flowing from the liver into the intestine [1,2]. This obstruction leads to the accumulation of bilirubin in the blood, resulting in jaundice [4]. The causes of obstructive jaundice can be categorized into benign and malignant etiologies [5]. Benign causes include choledocholithiasis (common bile duct [CBD] stones), benign strictures (which can result from chronic pancreatitis, previous surgeries, or inflammatory conditions), and biliary sludge [5]. Malignancies such as cholangiocarcinoma, pancreatic cancer, and metastatic tumors can lead to biliary obstruction [5]. Cholangiocarcinoma, in particular, is a primary malignancy of the bile ducts and is a significant cause of obstructive jaundice in the elderly [3-5]. Diagnosing bile duct obstruction in elderly patients requires a multimodal approach, integrating clinical evaluation, laboratory tests, and advanced imaging techniques [5]. A complete patient history and physical assessment are essential [5]. Symptoms such as scleral icterus, pruritus, right upper quadrant (RUQ) pain, fever, and weight loss can provide crucial clues [5]. However, these symptoms can be nonspecific and overlap with other conditions [5]. Hematological and serological tests are paramount for considering liver function and seeing the presence of jaundice [5,6]. Critical tests include serum bilirubin levels, liver enzymes, and indicators of inflammation such as C-reactive protein (CRP) [6]. Ultrasound is often the first-line imaging modality due to its availability and non-invasive nature [7]. Still, advanced imaging techniques such as computed tomography (CT) scans, magnetic resonance cholangiopancreatography (MRCP), and endoscopic ultrasound (EUS) provide detailed visualization of the biliary tree and surrounding structures [6]. ERCP is diagnostic and therapeutic, allowing for direct visualization, biopsy, and stent placement [6]. Managing obstructive jaundice in the elderly involves addressing the underlying cause of the obstruction and providing supportive care to manage symptoms [7]. Treatment may involve endoscopic or surgical obstruction removal [8]. Surgeons may indicate laparoscopic cholecystectomy in cases of gallstones after ERCP [8,9]. The treatment approach for malignant biliary obstructions typically includes surgery and additional therapies [7,8]. The Whipple procedure (pancreaticoduodenectomy) is a common surgical intervention for resectable pancreatic and periampullary cancers [7,8]. Adjuvant chemotherapy, typically with gemcitabine and cisplatin, reduces the risk of recurrence [8]. In cases where surgery is not feasible, palliative care, including stent placement and pain management, is essential to improve the quality of life [8]. This case report, combined with a minireview of relevant literature, aims to provide valuable insights into effectively managing this challenging condition, ultimately improving patient outcomes and quality of life.

## Case presentation

A 70-year-old man presented with a two-week history of progressive yellowish discoloration of the eyes, generalized itching, RUQ pain, and intermittent fevers, with a medical history of untreated HTN, type 2 DM, and a long-standing smoking habit, contributing to his high-risk profile. He appeared jaundiced and mildly distressed on examination, with scleral icterus and RUQ tenderness. Table 1 shows the different parameters of laboratory investigations of the patient.

**Table 1:** Laboratory findings CRP, C-reactive protein; AST, aspartate aminotransferase; ALT, alanine aminotransferase; ALP, alkaline phosphatase; ERCP, endoscopic retrograde cholangiopancreatography; TLC, total leukocyte count

Parameter	Pre-ERCP values	Post-ERCP values	Unit	Normal values
CRP	54.9	15.7	mg/L	<3 mg/L
Total bilirubin	12.9	2.9	mg/dL	0.1-1.2
AST	79	49	U/L	10-40
ALT	84	37	U/L	7-56
ALP	896	231	U/L	44-147
Creatinine	3.6	3.8	mg/dL	0.74-1.35
Urea	112	150	mg/dL	7-20
TLC	16.12	13.2	K/µL	4,000-11,000

**Figure 1:**
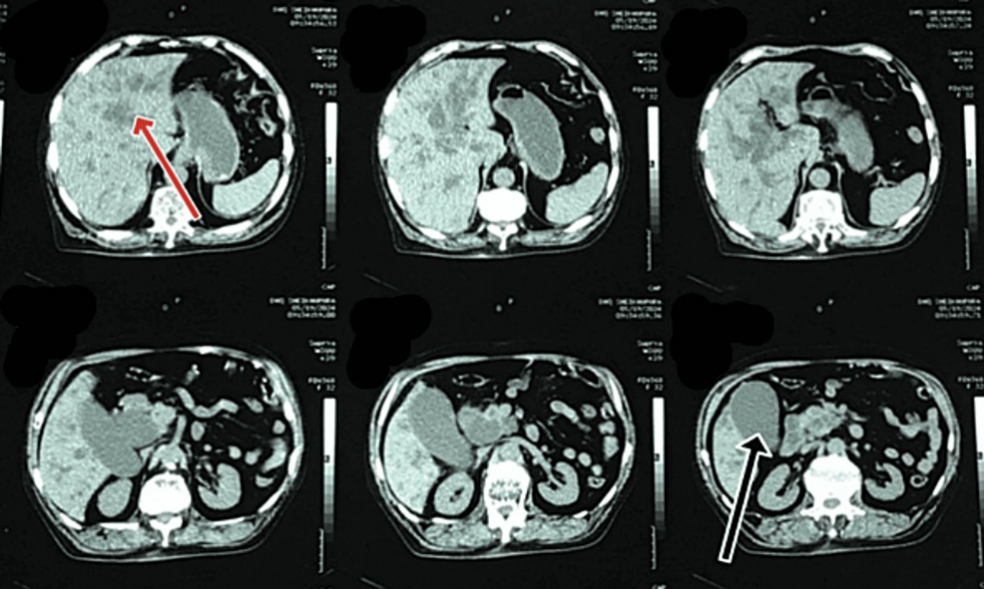
CT of the abdomen and pelvis without contrast showing moderate dilation of the CBD lumen proximal to IHBCs and significant distension of the GB lumen The red arrow shows the IHBC dilatation. The black arrow shows the distended GB. CT, computed tomography; CBD, common bile duct; IHBC, intrahepatic biliary channel; GB, gallbladder

These findings indicated an inflammatory and obstructive process within the biliary system. An abdominal ultrasound revealed a normal-sized liver (13.4 cm) with a normal parenchymal structure, a dilated portal vein (18 mm), and a mildly distended gallbladder (GB) full of sludge. Figure 1 shows the radiologic image of the non-contrast abdominal and pelvic CT scan, which indicated abrupt narrowing of the distal CBD, resulting in moderate dilation of the CBD lumen proximal to the intrahepatic biliary channels (IHBCs) and significant distension of the GB lumen.

**Figure 2:**
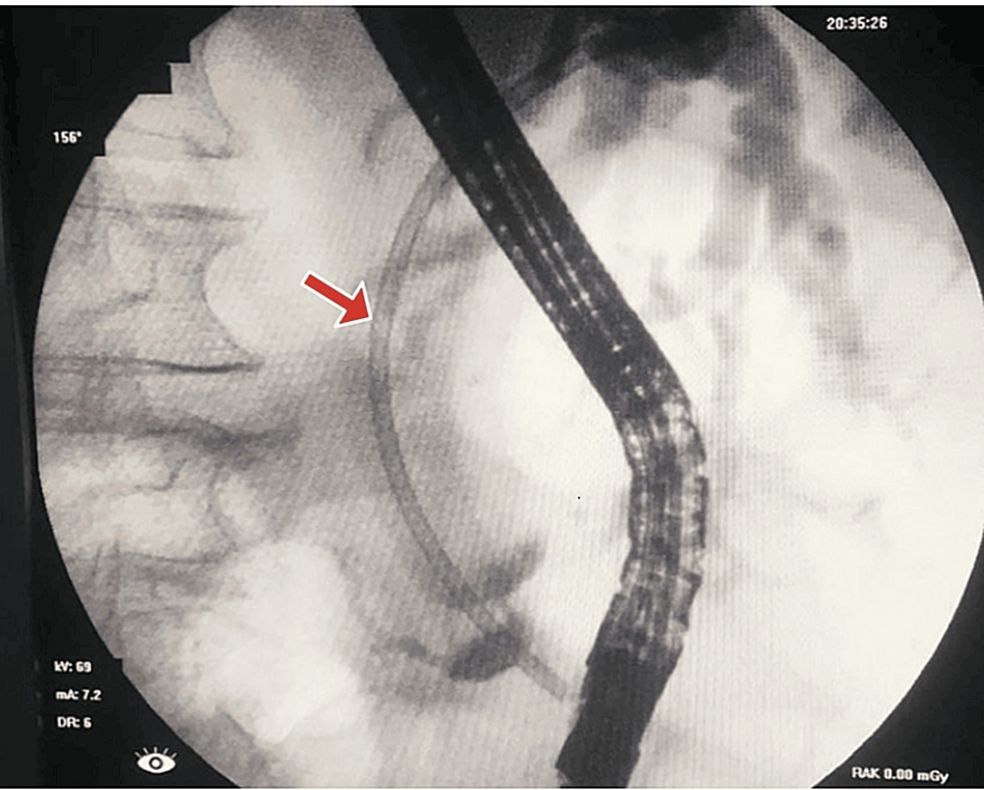
Cholangiogram taken during ERCP showing plastic stent inside CBD The red arrow shows the plastic stent placement inside CBD. ERCP, endoscopic retrograde cholangiopancreatography; CBD, common bile duct

The planned ERCP showed a limited view and revealed a normal esophagus, stomach, and duodenum. The ampulla appeared bulging and downlooking, making cannulation difficult and requiring more than 5 minutes. Initially, the pancreatic duct (PD) was cannulated, and dye was injected. Consequently, a 7Fr x 7cm plastic stent was placed into the PD following a limited sphincterotomy. After post-PD stenting, CBD was selectively cannulated into the right hepatic system, and dye injection showed grossly dilated CBD and IHBCs with a narrow segment at the distal end of the CBD. A 10Fr x 10cm plastic biliary stent was placed across the narrow segment, allowing free flow of bile and dye. ERCP showed a distal CBD stricture, suggestive of malignancy. The patient's CA 19-9 level was significantly elevated (>12,000), further supporting the suspicion of a malignant process. Figure 2 shows the cholangiogram, which revealed a grossly dilated CBD and IHBC dilatation with a narrow segment at the distal end of the CBD. A plastic stent, facilitating bile flow, can be seen in place.

**Figure 3:**
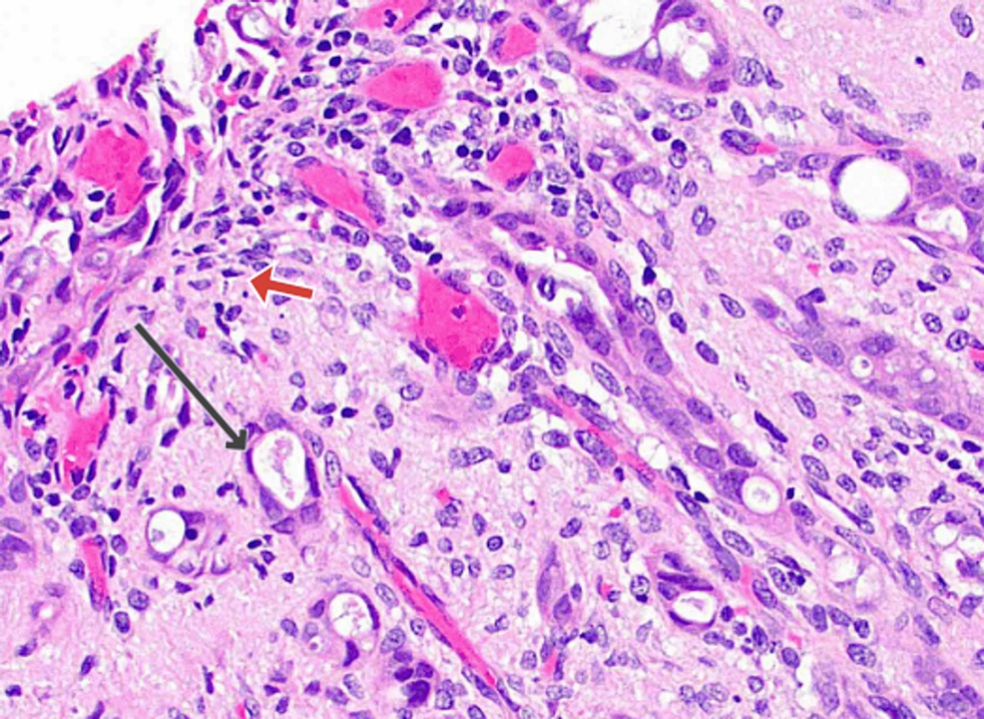
Histopathology of biopsy taken from distal CBD via ERCP brush cytology showing pleomorphic cells with inflammatory cells forming glands The black arrow indicates the glandular structure formed by adenocarcinoma cells. The red arrow shows the pleomorphic cell. CBD, common bile duct; ERCP, endoscopic retrograde cholangiopancreatography

A multidisciplinary team meeting recommended further investigation with a contrast-enhanced CT scan and repeat ERCP with brushing for tissue biopsy to confirm the diagnosis and guide management. In contrast, the CT of the chest, abdomen, and pelvis (CTCAP) revealed a persistent stricture of the distal CBD with no signs of local invasion or distant metastasis. The liver appeared slightly enlarged with mild hepatic steatosis and dilated IHBCs, but no focal lesions were detected. The pancreas was unremarkable, and there was no evidence of lymphadenopathy. The GB continued to show signs of sludge without any gallstones. The repeat ERCP with brushing provided tissue samples that, upon histopathological examination, confirmed the presence of well-differentiated adenocarcinoma of the distal CBD, graded as G1. Cytological analysis revealed malignant cells consistent with cholangiocarcinoma. Figure 3 shows the CBD histopathology, revealing glandular structures with associated inflammatory cells.

Based on the TNM staging system, the tumor was confined to the bile duct with muscle layer involvement, with no evidence of lymph node metastasis or distant metastasis, staging it as T1a N0 M0. Given the definitive diagnosis of distal CBD adenocarcinoma, a multidisciplinary team developed a comprehensive management plan. Considering the patient's overall health status and comorbidities, surgical resection was deemed a viable option. A Whipple procedure (pancreaticoduodenectomy) was recommended, involving the removal of the pancreatic head, the duodenum, part of the bile duct, the GB, and sometimes a portion of the stomach. This complex surgery is considered the standard treatment for resectable distal cholangiocarcinoma. Preoperative optimization included strict control of blood sugar levels and arterial pressure, nutritional support, and cessation of smoking to minimize surgical risks (pancreatic fistula, delayed gastric emptying, infection, bleeding, bile leak, anastomotic leak, pulmonary and cardiovascular complications).

Additionally, a multidisciplinary team approach was employed to ensure comprehensive preoperative assessment and preparation. No major postsurgical complication was observed in the patient except for minor bleeding. Postoperative adjuvant therapy with chemotherapy was planned to lower the chances of recurrence and enhance long-term survival. Postoperative adjuvant treatment with gemcitabine and cisplatin was intended to reduce the elevated chance of recurrence and benefit long-term survival, with gemcitabine administered at 1,000 milligrams per square meter on days 1, 8, and 15 of a 28-day cycle, along with cisplatin at 25 milligrams per square meter on days 1 and 8 of a 28-day cycle, for six to eight cycles. Regular follow-ups every three months for the first two years, then every six months up to five years, were scheduled to assess treatment response and manage potential complications. The patient experienced a favorable overall outcome, with effective treatment response and manageable post-surgical complications, contributing to an improved quality of life and prognosis. The comprehensive management plan aims to optimize outcomes and improve the quality of life, with adjustments based on his response and emerging clinical issues.

## Discussion

This case depicts the complexities of diagnosing and managing obstructive jaundice in elderly patients with multiple comorbidities. Obstructive jaundice, characterized by the impeded flow of bile from the liver to the duodenum, can result from a variety of underlying conditions, including both benign and malignant etiologies. In elderly patients, additional health conditions such as DM and HTN complicate the clinical picture [8,9]. These comorbidities, along with factors such as long-term smoking, not only increase the risk of CVD but also heighten the risk of gastrointestinal malignancies, including cholangiocarcinoma [10,11]. The differential diagnosis for obstructive jaundice is broad, necessitating a comprehensive diagnostic approach [1,2]. Initial imaging typically involves ultrasound due to its accessibility and effectiveness in detecting biliary dilatation and gallstones [3]. In this case, an abdominal ultrasound revealed a dilated portal vein and a distended GB full of sludge, but the exact cause of obstruction was not identified. Further evaluation with CT and ERCP was necessary to elucidate the underlying pathology. ERCP remains a cornerstone in diagnosing and treating biliary obstructions, offering diagnostic and therapeutic capabilities [3,4]. In this case, ERCP was instrumental in visualizing the bile ducts, diagnosing the CBD stricture, and facilitating biliary drainage through stenting. The elevated CA 19-9 level and the histopathological confirmation of well-differentiated adenocarcinoma underscored the malignant nature of the obstruction. Management of obstructive jaundice is highly dependent on the underlying cause [4,5]. Benign causes, such as choledocholithiasis, are often managed with endoscopic or surgical interventions to remove obstructions and restore bile flow [11,12].

In contrast, malignant obstructions typically require a combination of surgical resection and adjuvant therapies [12,13]. The Whipple procedure (pancreaticoduodenectomy) is the gold standard for resectable periampullary and pancreatic cancers despite its significant risks, especially in elderly patients with multiple comorbidities [12,13]. This complex surgery involves removing the pancreatic head, duodenum, part of the bile duct, the GB, and sometimes a portion of the stomach [13]. Despite its complexity, it offers the best chance for long-term survival in patients with resectable cholangiocarcinoma [13]. In this case, preoperative optimization was crucial due to the patient's comorbidities, ensuring strict control of blood sugar levels and arterial pressure, along with nutritional support and smoking cessation. The planned adjuvant therapy with gemcitabine and cisplatin aims to reduce recurrence risk and improve survival outcomes. Regular follow-ups are essential to monitor treatment response and manage potential complications. This case highlights the necessity for a multidisciplinary approach in managing complex cases of obstructive jaundice. Collaboration among healthcare providers ensures comprehensive evaluation and tailored management plans that address curative and palliative needs. For non-surgical candidates due to advanced disease or poor health status, palliative care focuses on symptom management and maintaining quality of life, with interventions such as endoscopic or percutaneous stenting being crucial for relieving jaundice and preventing cholangitis [11-13]. The differential diagnosis for obstructive jaundice also includes conditions such as bile duct strictures post-surgery, parasitic infections such as liver flukes, and autoimmune diseases such as IgG4-related sclerosing cholangitis [13]. Each condition requires a tailored diagnostic and management approach, emphasizing the importance of thorough evaluation and personalized care [13].

In conclusion, this case emphasizes the importance of integrating advanced diagnostic tools, a multidisciplinary approach, and balancing curative and soothing strategies to achieve the best possible outcomes for patients with complex medical backgrounds. Ongoing research and continuous advancements in minimally invasive techniques and therapeutic options are crucial to enhancing care and outcomes in the management of obstructive jaundice, particularly in elderly patients with multiple comorbidities.

## Conclusions

This case study emphasizes the challenges and complexities in exploring the diagnosis and managing obstructive jaundice in elderly patients with multiple comorbidities. The necessity for a broad differential diagnosis, including both benign and malignant causes, underscores the need for a comprehensive diagnostic and management approach. Advanced imaging and endoscopic techniques, particularly ERCP, are essential for accurate diagnosis and effective intervention. In this case, a CBD stricture was diagnosed as adenocarcinoma, prompting a multidisciplinary treatment plan involving a Whipple procedure and adjuvant chemotherapy. The case underscores the importance of a personalized care approach, integrating advanced diagnostics and interdisciplinary collaboration, to achieve optimal outcomes for elderly patients with complex medical conditions. It also emphasizes the value of continuous advancements in minimally invasive techniques and therapeutic options, essential for improving patient care and outcomes in managing obstructive jaundice. This case contributes to the medical field by illustrating the critical need for tailored, patient-specific strategies, and the potential benefits of a multidisciplinary approach in managing challenging cases of obstructive jaundice.
